# Accuracy of direct genomic values in Holstein bulls and cows using subsets of SNP markers

**DOI:** 10.1186/1297-9686-42-37

**Published:** 2010-10-16

**Authors:** Gerhard Moser, Mehar S Khatkar, Ben J Hayes, Herman W Raadsma

**Affiliations:** 1 Dairy Futures Cooperative Research Centre (CRC), Australia; 2 ReproGen - Animal Bioscience, Faculty of Veterinary Science, University of Sydney, 425 Werombi Road, Camden NSW 2570, Australia; 3 Biosciences Research Division, Department of Primary Industries Victoria, 1 Park Drive, Bundoora 3083, Australia

## Abstract

**Background:**

At the current price, the use of high-density single nucleotide polymorphisms (SNP) genotyping assays in genomic selection of dairy cattle is limited to applications involving elite sires and dams. The objective of this study was to evaluate the use of low-density assays to predict direct genomic value (DGV) on five milk production traits, an overall conformation trait, a survival index, and two profit index traits (APR, ASI).

**Methods:**

Dense SNP genotypes were available for 42,576 SNP for 2,114 Holstein bulls and 510 cows. A subset of 1,847 bulls born between 1955 and 2004 was used as a training set to fit models with various sets of pre-selected SNP. A group of 297 bulls born between 2001 and 2004 and all cows born between 1992 and 2004 were used to evaluate the accuracy of DGV prediction. Ridge regression (RR) and partial least squares regression (PLSR) were used to derive prediction equations and to rank SNP based on the absolute value of the regression coefficients. Four alternative strategies were applied to select subset of SNP, namely: subsets of the highest ranked SNP for each individual trait, or a single subset of evenly spaced SNP, where SNP were selected based on their rank for ASI, APR or minor allele frequency within intervals of approximately equal length.

**Results:**

RR and PLSR performed very similarly to predict DGV, with PLSR performing better for low-density assays and RR for higher-density SNP sets. When using all SNP, DGV predictions for production traits, which have a higher heritability, were more accurate (0.52-0.64) than for survival (0.19-0.20), which has a low heritability. The gain in accuracy using subsets that included the highest ranked SNP for each trait was marginal (5-6%) over a common set of evenly spaced SNP when at least 3,000 SNP were used. Subsets containing 3,000 SNP provided more than 90% of the accuracy that could be achieved with a high-density assay for cows, and 80% of the high-density assay for young bulls.

**Conclusions:**

Accurate genomic evaluation of the broader bull and cow population can be achieved with a single genotyping assays containing ~ 3,000 to 5,000 evenly spaced SNP.

## Background

In genomic selection (GS), selection decisions are made on genomic breeding values predicted from high-density single nucleotide polymorphic (SNP) markers. In dairy cattle, GS has the potential to double the rate of genetic gain to that of traditional breeding schemes due to a substantial reduction in generation intervals and increased selection intensities [1,2]. Significant additional gains in GS schemes could be made if cows to breed sires and cows to breed cows were selected on genomic breeding values [1]. Another benefit of genotyping cows may be lower rates of inbreeding: according to Daetwyler et al. [3], the use of GS can be expected to decrease the rate of inbreeding relative to conventional selection using BLUP breeding values, this effect will be greatest when larger numbers of both cows and potential sires are genotyped [4].

At the current price, high-density SNP genotyping assays are limited to applications involving elite sires and dams. An alternative is to use a more cost-effective low-density assay for genotyping more animals from the population. As shown for a single trait by Weigel et al. [5], a low-density assay comprising selected SNP can deliver a substantial portion of the gain of a high-density assay, possibly for a fraction of the price. However, the use of such a low-density array may still be limited if multiple traits require so many SNP that their genotyping cost is similar to the cost of a high-density chip.

The utility of low-density arrays will depend in part on the genetic architecture of the target trait. In GS, prediction equations are derived from a training set, where animals are phenotyped and genotyped to predict breeding values based only on the genotype information of evaluation animals. This requires that the markers are in sufficient LD with the QTL and simulation studies have shown that accuracy of genomic predictions increases as LD increases [6-10]. In the ideal case where every QTL is in perfect LD with a single marker and where a limited number of QTL with large effects account for the genetic variation, the maximum accuracy could be obtained with very few markers. However, there is increasing evidence that most complex traits are affected by very many QTL with a small effect (e.g. height in humans, [11-14]). This would imply that the training population would need to be genotyped with a high-density SNP panel in order to capture the effects of all QTL. Selecting individual SNP from high-density genotype data is complicated because the multicollinearity between SNP, i.e. two or more SNP in high but not complete LD, makes it difficult to identify 'important' SNP, as each SNP masks a part of the effect of other SNP and a single marker might be in LD with several QTL.

Utility of SNP subsets will also be affected by the relationship of the selection candidates to the training set. Although genomic predictions rely on LD between SNP and QTL, this LD can operate or be interpreted at a number of levels. In addition to population level LD, simulation studies and empirical data have demonstrated that the accuracy of prediction depends on the relatedness between animals in the training and evaluation populations [10,15,16]. At the extreme, even in the absence of LD between markers and QTL, markers can predict family relationships between animals. If animals in the training and evaluation data share DNA segments from a small number of ancestors, relatively few markers are required to trace the segments shared between related animals separated by only a few generations. A low-density assay of evenly spaced SNP might then provide sufficient accuracies of prediction of evaluation animals, as long as the information content of the subset of SNP is sufficient to estimate effects of distinct haplotypes.

The objective of this study was to evaluate the use of low-density SNP genotyping assays to predict the direct genomic value (DGV) of bulls and cows for commercially important traits in Holstein-Friesian dairy cattle. The impact of two analysis methods, the number of SNP needed for accurate DGV prediction, as well as strategies for SNP selection were explored.

## Methods

### Phenotype and genotype data

Phenotype and genotype data were available on 2,144 Holstein-Friesian bulls and 510 Holstein-Friesian cows. The traits analysed included milk production traits (milk yield, fat yield, protein yield, fat percentage and protein percentage), an overall confirmation trait (overall type), survival index, Australian Profit Ranking (APR) and Australian Selection index (ASI). The ASI is an index given by (3.8 × protein ABV) + (0.9 × fat ABV) - (0.048 × milk ABV), APR is given by (3.8 × protein ABV) + (0.9 × fat ABV) - (0.048 × milk ABV) + (1.2 × milking speed ABV) + (2.0 × temperament ABV) + (3.9 × survival ABV) + (0.34 × cell count ABV) - (0.26 × live weight ABV) + (3.0 × daughter fertility), whereas survival is given by (0.5 × likeability) + (1.8 × overall type) + (3.0 × udder depth) + (2.2 × pin set).

Phenotype information was provided by the Australian Dairy Herd Improvement Scheme (ADHIS, http://www.adhis.com.au). The phenotypes used were deregressed breeding values (DRBV) for protein percentage, fat percentage, ASI, APR and survival, and daughter trait deviations (DTD) for protein yield, fat yield, milk yield and overall type. The deregression procedure removed the contribution of relatives other than daughters to the breeding values, as detailed in [17]. For cows, trait deviations (TD) were available for protein yield, fat yield, milk yield and overall type, but no DRBV information was available for the other traits.

SNP genotypes were derived from the Illumina BovineSNP50 BeadChip (Illumina Inc., San Diego, USA). After quality control [18] and omitting SNP located on the sex chromosomes a total of 42,576 markers remained for the analysis.

### Training and validation sets and accuracy of DGV

**Figure 1:**
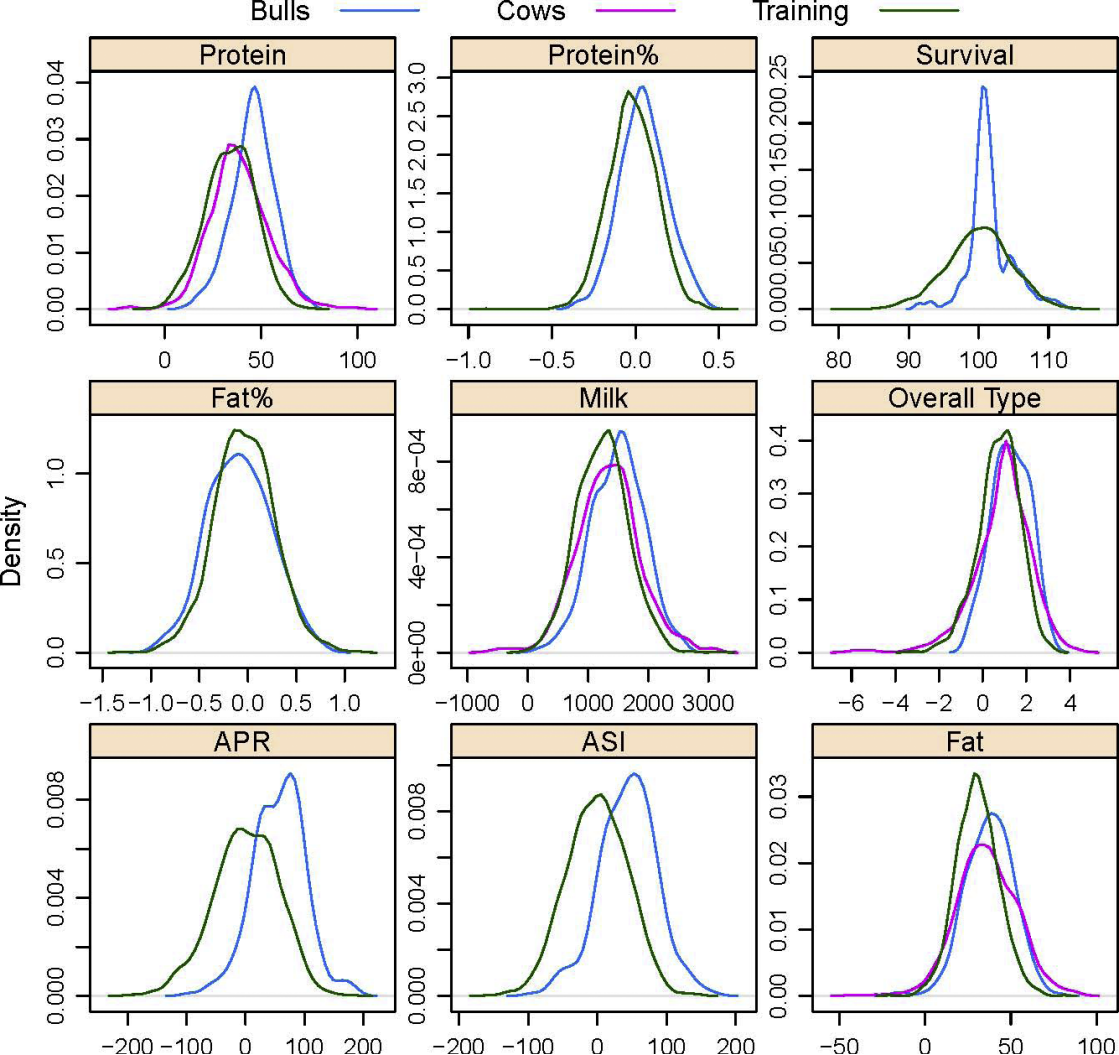
**Density plots of phenotypes in the training set and the validation sets**.

The 2,144 bulls were divided in a training data set of 1,847 bulls born between 1955 and 2004 and a validation set of 297 young bulls born between 2001 and 2004, which represented progeny test teams for 2007, 2008 and 2009. A second validation set included 510 cows born between 1992 and 2004. Table 1 gives the number of animals in training and test sets and the number of records contributing to the phenotypes per animal. Of the 297 young bulls in the bull validation set, 240 (80.8%) were sired by bulls in the training set, whereas 473 (92.7%) of the cows had their sire in the training set. The correlation coefficient between predicted DGV and realized DRBV, DTD or TD was used as the measure of accuracy of DGV prediction. The distribution of traits in the training and validation set is shown in Figure 1.

**Table 1:** Number of animals in training and validation sets and median number of records contributing to the phenotype per animal

		Training set			Validation sets					
			
Trait	**Phenotype**^**a**^	Bulls	**Records**^**b**^		Bulls	**Records**^**b**^		Cows	**Records**^**c**^	
Protein, Fat, Milk	DTD, TD	1845	107	(82, 165)	297	71	(59, 87)	510	5	(3, 6)
Overall Type	DTD, TD	1314	35	(23, 57)	89	36	(29, 46)	313	1	(1, 1)
Protein%, Fat%, ASI	DRBV	1845	107	(82, 165)	297	71	(59, 87)			
APR	DRBV	1828	73	(54, 106)	295	32	(27, 49)			
Survival	DRBV	1847	39	(29, 58)	227	4	(4, 29)			

^a ^DTD: daughter trait deviations for bulls; TD: trait deviations for cows; DRBV: deregressed breeding value

^b ^Median number of phenotyped daughters per bull, 25^th ^and 75^th ^percentile in parentheses

^c ^Median number of lactations per cow, 25^th ^and 75^th ^percentile in parentheses

### Calculation of DGV

Prediction equations for each trait were derived from the training set by either ridge regression [19,20] or partial least squares regression [9,20,21] and then combined with the genotype data to predict DGV for the validation animals:

$$DGV=X\hat{b}\text{,}$$

where DGV is the vector of direct genomic values estimated with the marker genotypes, **X **is an incidence matrix that relates genotypes to individuals, and $\hat{b}$ is the vector of SNP effects which is estimated by either one of the two methods described below.

### Ridge regression (RR)

Regression coefficients are obtained from the solution of the mixed model equations

$$\left[\begin{array}{c} \hat{\mu }\\ \hat{b} \end{array}\right]={\left[\begin{array}{cc} N & 1^{/}X\\ X^{/}1 & X^{/}X+I\lambda \end{array}\right]}^{-1}\left[\begin{array}{c} 1^{/}y\\ X^{/}y \end{array}\right],$$

where *N *is the number of training animals, **y **is a vector of phenotypes, $\hat{\mu }$ is an unknown constant, **X **is a (*N *× *p*) matrix of genotypes encoded as 0 (homozygote), 1 (heterozygote) or 2 (other homozygote), ${\hat{b}}^{/}=\left[{\hat{\beta }}_{1},\ldots ,{\hat{\beta }}_{p}\right]$ is a vector of SNP effects, and **I **is a *p *× *p *identity matrix. The penalty term *λ*, which is the same for all SNP, overcomes the problem of ill-conditioning when multicollinearity among columns in **X **causes **X'X **to be singular, or nearly so. The system of equations was solved iteratively by the preconditioned conjugate gradient method [22]. The 10-fold cross-validation procedure described in Moser et al. [20], with golden segment search [23], was used to locate the optimal *λ *within a given range. RR is equivalent to the BLUP method of Meuwissen et al. [6] and Habier et al. [15], which assumes that regression coefficients are independent random draws from a common normal distribution. Under the BLUP model, *λ *= σ*^2^_e _/*σ*^2^_g_*, where σ*^2^_e _*is the residual variance and σ*^2^_g _*the genetic variance.

In RR, the contribution of each bull can be weighted according to the number of daughters contributing to the phenotype. However, reliabilities of the phenotypes expressed as 'equivalent daughter contributions' were uniformly high, with small differences between the majority of training bulls, and weighting the contributions of bulls had no impact on the accuracy of DGV for method RR (results not shown).

### Partial least squares regression (PLSR)

The main idea of PLSR is to build orthogonal components (called 'latent components') from the original genotype matrix **X**. A PLSR component ***t ***= **X*w ***is a linear combination of the SNP that have maximal covariance with the response vector, under the additional assumption that components are mutually orthogonal [24]. Subsequently, **y **is regressed on the linear combinations of markers.

Different algorithms to extract the latent components and to obtain regression coefficients $\hat{b}$ exist. We implemented PLSR using an algorithm described in [25]. The optimal model complexity (i.e. number of latent components), was estimated by ten fold cross-validation [20]. Note that the PLSR regression coefficients differ from the ordinary least squares regression coefficients and the RR regression coefficients. The magnitude of the PLSR regression coefficients can be used to determine the relative influence of each SNP on the model and to select relevant SNP [26].

### SNP selection

The absolute magnitude of the regression coefficients was used to determine which SNP are most influential in the training data set. To select subsets of markers, all 42,576 SNP were ranked by their absolute value of $\hat{b}$. The ranking of SNP was derived using a backward elimination procedure. The process started with a model including the complete set of 42,576 SNP. Subsequently in each step, a fraction of SNP with the smallest absolute value of the regression coefficients was dropped from the SNP list and the regression coefficients were recomputed. This re-computation is important as the regression coefficient of an individual SNP can strongly depend on other SNP that are in LD with the SNP of interest. The optimal model complexity (i.e. number of latent components) for PLSR and the value of *λ *for RR was estimated at each step by cross-validation.

In detail, we first fitted models including all 42,576 SNP. In the first iteration 40,000 SNP with the highest absolute value of the regression coefficient were retained in the SNP list. The number of SNP subsequently dropped in each iteration was 2,000 for subsets of up to 10,000 SNP, 500 SNP for subsets of up to 1,000 SNP, 100 SNP for subsets of up to 300 SNP and 20 SNP for subsets of up to 100 SNP.

Four alternative strategies of SNP subset selection were compared. Under strategy 1, separate subsets including the highest ranked SNP for each individual trait were created. Strategies 2-4 used a single subset of evenly spaced SNP. To select a subset of *n *evenly spaced SNP, we divided the total length of the autosomes into *n *intervals flanked by two markers to give segments of approximately equal length. Chromosome lengths and SNP positions were based on the physical map of cattle genome assembly Btau 4.0. Subsequently, the highest ranked SNP for ASI (strategy 2), APR (strategy 3) or the SNP with the highest minor allele frequency (MAF, strategy 4) in each segment, was added to the subset. Using the same subset of SNP, a model was then fitted for each trait to derive the prediction equations. Subsets of evenly spaced SNP were generated for sets including between 100 and 5,000 SNP. The accuracy of DGV obtained using a subset of SNP was compared to the accuracy from the analysis of all 42,576 SNP.

## Results

### Accuracy of DGV using trait-dependent SNP subsets derived with RR and PLSR

**Figure 2:**
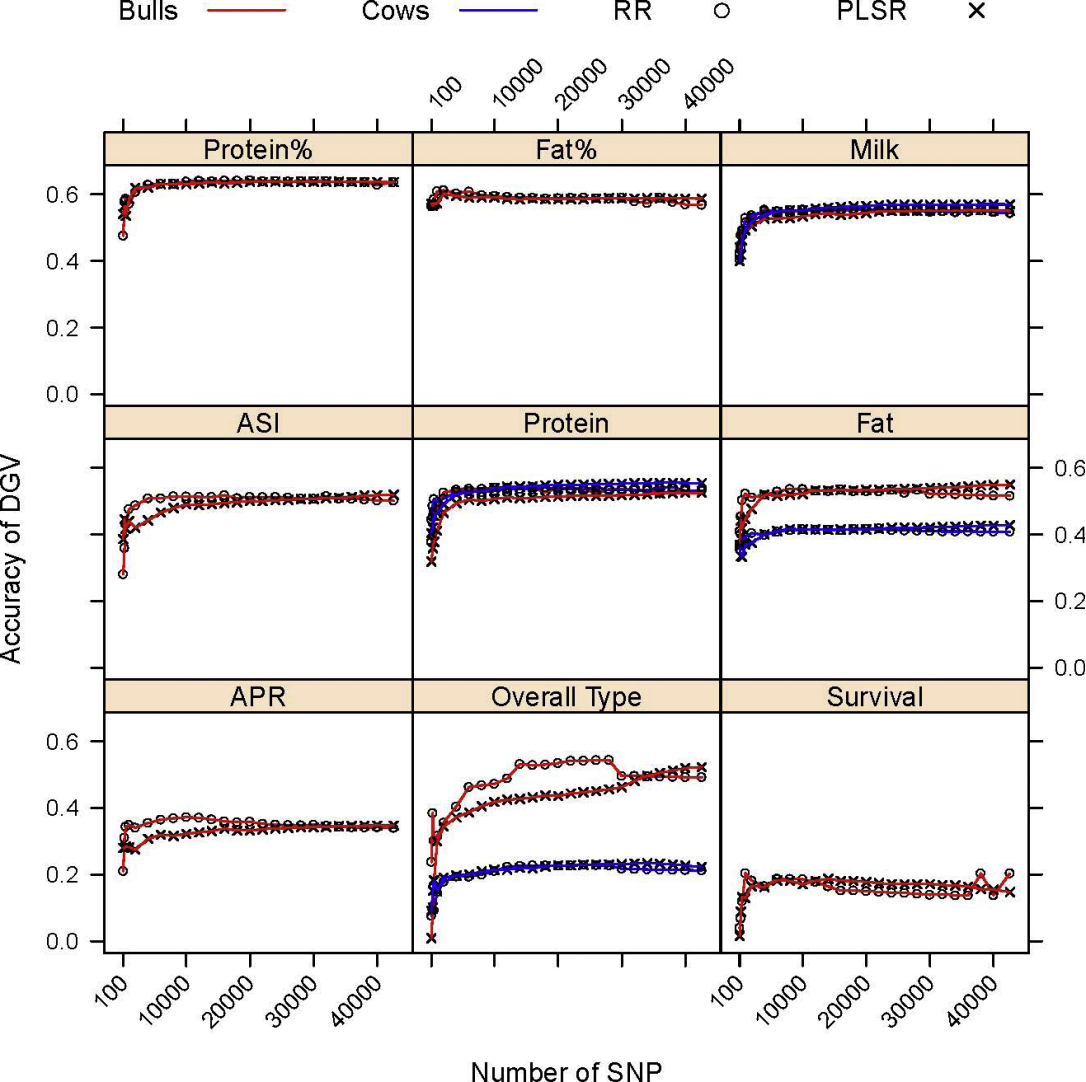
**Accuracy of DGV of bulls and cows using subsets of the highest ranked SNP obtained by RR and PLSR**.

Accuracy of DGV predictions in validation sets of young bulls and cows using all 42,576 SNP and subsets including the highest ranked SNP for each trait are shown in Figure 2. Accuracy of DGV was computed as the correlation between DGV and the phenotype. Accuracy of prediction for protein percentage, fat percentage, ASI, APR and survival could not be computed for cows, because phenotypes for these traits were not available. Accuracy of DGV prediction from the analysis of all 42,576 SNP ranged from 0.15 to 0.64 for RR and 0.20 to 0.64 for PLSR in the validation set of bulls, and from 0.22 to 0.57 for RR and from 0.21 to 0.54 for PLSR in the validation set of cows (Figure 2). The largest difference between the bull and cow validation sets was obtained for the overall type trait, with the accuracy of DGV for cows being approximately half that of bulls, whereas for protein and milk yield the accuracies of DGV prediction between bulls and cows were almost identical (Figure 2).

Overall, predictions by RR were slightly more accurate for larger SNP subsets but less accurate for smaller SNP subsets compared to PLSR. As shown in Table 2, the differences in accuracy between both methods, with respect to the highest correlation obtained for an individual trait, were negligible. The highest accuracy for PLSR was obtained with models that contained considerably fewer SNP than the high-density assay, whereas the RR model with the highest accuracy included almost all SNP, with the exception of survival and fat percentage. In the case of PLSR, the highest accuracy for cows was achieved with models containing more SNP compared to bulls (Table 2). Depending on the trait, accuracies of PLSR were 2 to 12% higher than those for RR for subsets including 5,000 or less SNP [see Additional file 1].

**Table 2:** Maximum accuracy of DGV of cows and bulls derived by RR and PLSR

	Bulls				Cows			
		
	RR		PLSR		RR		PLSR	
			
Trait	SNP	Accuracy	SNP	Accuracy	SNP	Accuracy	SNP	Accuracy
Protein	42,576	0.52	9,000	0.54	38,000	0.56	36,000	0.54
Fat	42,576	0.55	9,000	0.54	42,576	0.43	22,000	0.42
Milk	36,000	0.55	4,500	0.56	40,000	0.57	18,000	0.56
Overall Type	42,576	0.52	28,000	0.54	34,000	0.23	24,000	0.23
Protein%	32,000	0.64	20,000	0.64				
Fat%	3,500	0.60	900	0.62				
ASI	42,576	0.52	16,000	0.52				
APR	42,576	0.35	10,000	0.37				
Survival	14,000	0.19	1,000	0.20				

The panels in Figures 2 are ordered from high to low heritable traits (left-right, top-bottom) based on reported heritability estimates [27,28]. Heritability of APR and ASI was assumed to be intermediate between production traits and survival. Figure 2 shows a strong relationship between the accuracy of prediction of DGV and the heritability of the trait. Predictions of production traits with a higher heritability, such as protein percentage (h^2 ^= 0.56), fat percentage (h^2 ^= 0.52), and milk yield (h^2 ^= 0.28), were more accurate than predictions of traits with a lower heritability, such as overall type (h^2 ^= 0.18) and survival (h^2 ^= 0.03).

### Accuracy of DGV using low-density assays depending on the method of SNP selection

Figure 2 shows a consistent trend in the accuracy of DGV when the SNP density decreased from 42,576 to approximately 1,000 SNP using trait-depended subsets of SNP. When SNP density exceeded 1,000 SNP the accuracy of DGV reached a plateau, and increases in accuracy with increasing number of SNP were marginal or fluctuated around the maximum accuracy (Table. 2). This plateau in accuracy of DGV was consistent in both bulls and cows (Figure 2). At densities below 1,000 SNP accuracies declined relatively rapidly, subsets of 100 SNP consistently showed the lowest accuracy within the range examined here (Figure 2).

**Figure 3:**
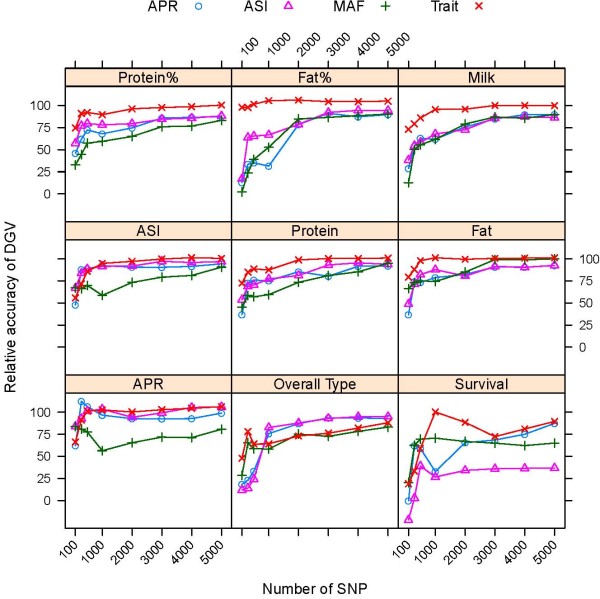
**Accuracy of DGV of bulls using low-density assays depending on the method of SNP selection**. Accuracy of prediction is shown as percentage of the accuracy obtained with 42,576 SNP for subsets including the highest ranked SNP (Trait), subsets of evenly spaced SNP including the highest ranked SNP for ASI (ASI), APR (APR) or SNP with highest minor allele frequency (MAF) obtained by PLSR

**Figure 4:**
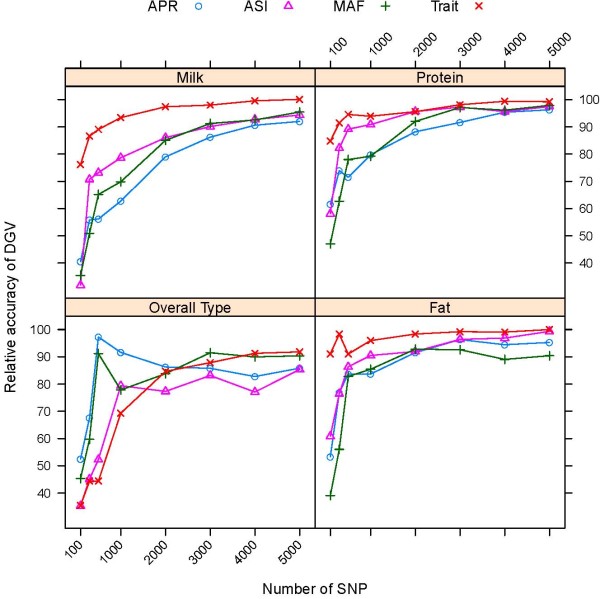
**Accuracy of DGV of cows using low-density assays depending on the method of SNP selection**. Accuracy of prediction is shown as percentage of the accuracy obtained with 42,576 SNP for subsets including the highest ranked SNP (Trait), subsets of evenly spaced SNP including the highest ranked SNP for ASI (ASI), APR (APR) or SNP with highest minor allele frequency (MAF) obtained by PLSR

Results showing the accuracy of DGV using subsets of SNP selected by each of the four strategies are restricted to the analyses of subsets of 100, 300, 500, 1,000, 3,000 and 5,000 SNP. To limit redundancy, results from the analyses using RR are not presented in detail, but RR performed very similar to PLSR as shown in Figure 2. Relative accuracies of prediction are expressed as percentage of the accuracies obtained with 42,467 SNP and are shown in Figure 3 for bulls and Figure 4 for cows.

When the number of SNP in the subset was 1,000 or larger, using trait-specific subsets gave higher accuracies than using a common subset of SNP in both validation sets, with the exception of overall type for both bulls and cows (Figure 3 and 4). In addition, the rate of decrease in accuracy, with respect to the size of the subset, was much more rapid for evenly spaced SNP than for trait-dependent SNP. The rate of decrease in accuracy tended to be lower for production traits, which have a higher heritability than traits related to fitness. Predictions based on at least 1,000 or 3,000 SNP appeared to be very robust to how SNP were selected, but were very sensitive when the subset included fewer SNP.

For the overall type trait, subsets including more than 1,000 of the highest ranked SNP for the trait gave lower accuracies than evenly spaced SNP selected for ASI and APR, which might be due the smaller number of training records available for this trait. All subsets containing less than 500 SNP performed poorly for survival, which has a low heritability (h^2 ^= 0.03), particularly subsets of SNP selected for APR and ASI.

The relative accuracy of prediction using low-density assays across the nine traits available for bulls and the four traits available for cows is given in Table 3. Higher relative accuracies were found for cows compared to bulls, which is partly due to the fact that production traits with higher DGV accuracies contributed more to the average of cows. Subsets including the highest ranked SNP for each trait outperformed a single subset of common SNP, which is expected as a common SNP subset of the same size will not include the highest ranked SNP for each trait, with exceptions for bulls for subsets of 3,000 or 5,000 SNP selected for the index APR or of 3,000 SNP selected for the index ASI. However, the gain in accuracy using subsets of the highest ranked SNP over a common set of SNP was small when at least 3,000 SNP were used. A subset containing 5,000 evenly spaced SNP selected for APR captured 92% of the accuracy of the high-density assay in both bulls and cows, compared to average relative accuracies of 89% in bulls and 98% in cows, when using trait-specific subsets with the highest ranked SNP for each trait. Irrespective of the method of SNP selection, subsets containing 3,000 SNP provided more than 90% of the accuracy that could be achieved with a high-density assay for cows, and 80% for young bulls.

**Table 3:** Summary of accuracy of DGV using low-density assays derived by PLSR

Test set	SNP selection	Number of SNP					
		
		5,000	3,000	1,000	500	300	100
Bulls		Trait-specific assay					
		89	85	84	78	72	59
		Common assay of evenly spaced SNP					
	ASI	88	86	68	67	65	32
	APR	92	86	68	67	65	32
	MAF	86	80	61	62	58	40
							
Cows		Trait-specific assay					
		98	96	88	80	80	72
		Common assay of evenly spaced SNP					
	ASI	94	92	85	75	69	47
	APR	92	90	79	77	69	52
	MAF	94	93	78	79	57	42

Accuracy of prediction is shown as percentage of the accuracy obtained with 42,576 SNP, averaged over nine traits for bulls and four traits for cows.

**Figure 5:**
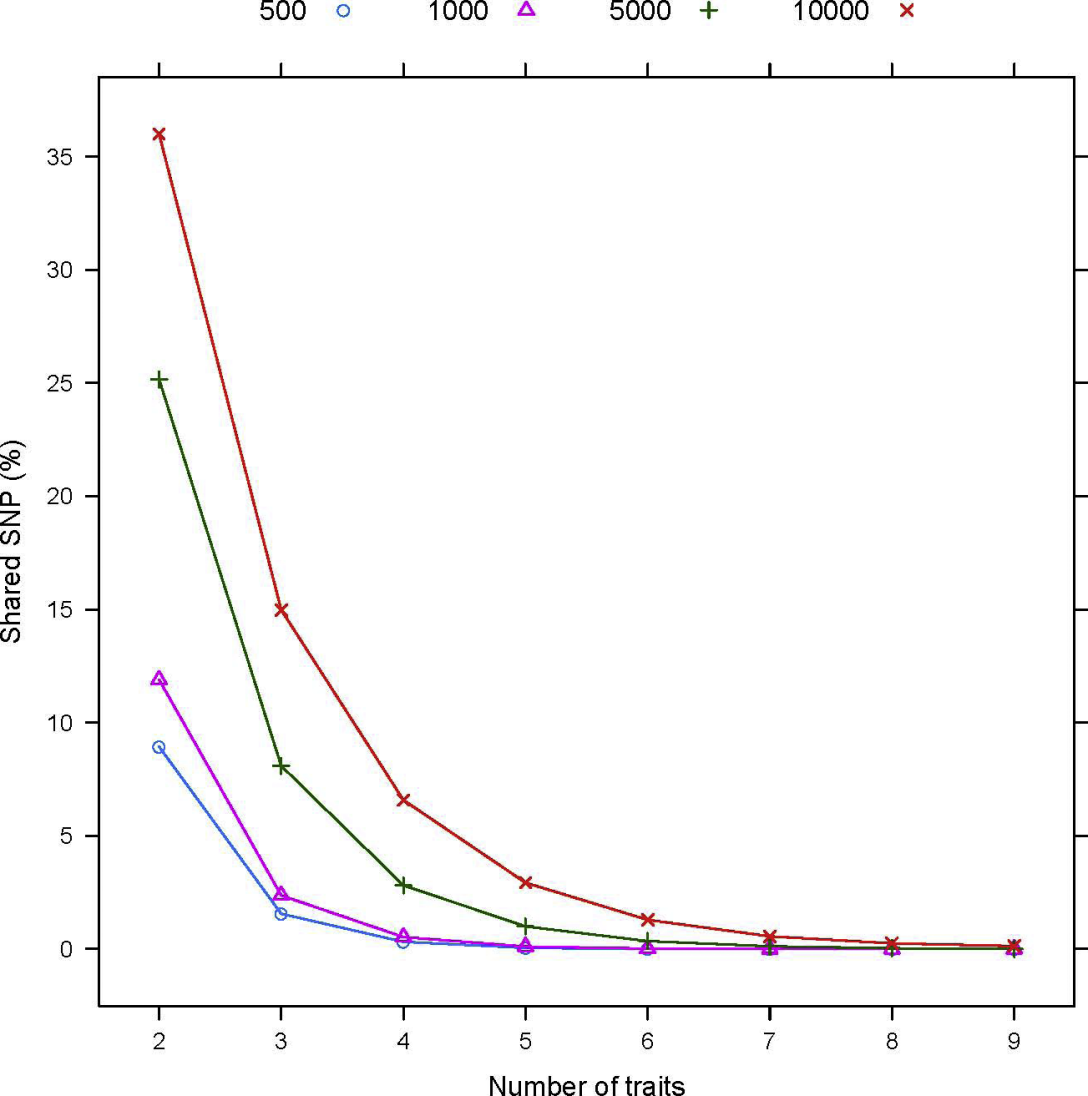
**Percentage of the highest ranked SNP that are shared between sets of traits**. Percentage of SNP that are shared between all combinations of sets of traits for subsets including 500, 1,000, 5,000 or 10,000 SNP

Figure 5 shows the percentage of SNP that were shared between combinations of traits, with the number of traits ranging from two to nine. The average number of SNP shared between any two traits was 35% for subsets of 10,000 SNP and dropped to under 10% for subsets of 500 SNP. As the number of traits increased, the number of SNP in common between traits decreased rapidly. Only 0.13% of the 10,000 highest ranked were in common among all nine traits, and no SNP was in common for all traits for subsets of 5,000 SNP. In general, a larger proportion of SNP was shared between index traits and the traits included in the index (results not shown). For example, approximately 60% of the 5,000 highest ranked SNP for ASI were also included in the subset for APR, but less than 20% of those SNP were included in the subsets for fat percentage and protein percentage.

### Accuracy of DGV for bulls and cows with or without genotyped sires in the training set

**Figure 6:**
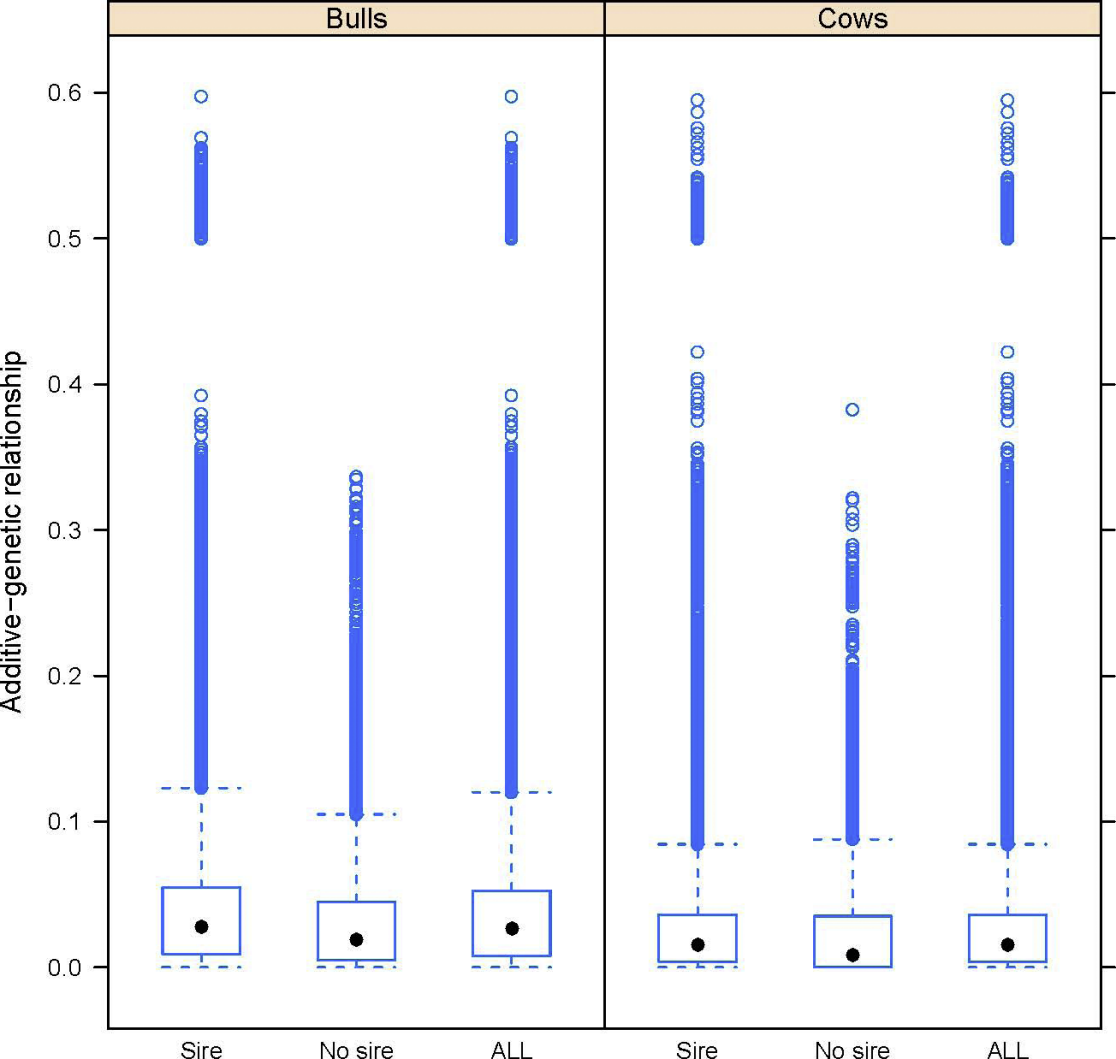
**Box-and-whiskers plots of additive-genetic relationships between training and validation animals**. Additive-genetic relationships calculated from pedigree are shown between training and validation animals (ALL) and between training animals and validation animals whose sires were included (Sire) or were not included (No Sire) in the training data

**Figure 7:**
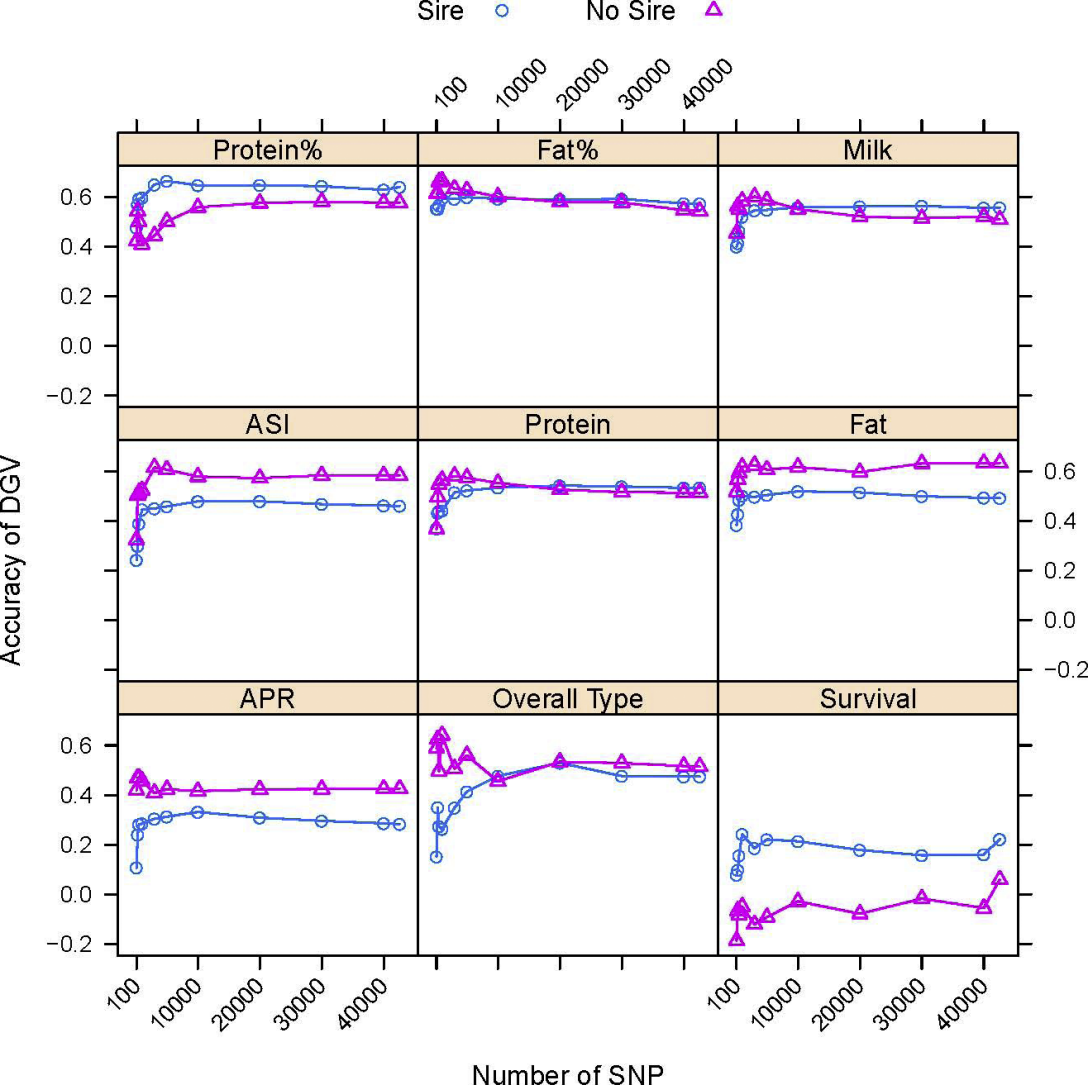
**Accuracy of DGV of bulls with or without sire in the training data using trait-specific SNP subsets**. Accuracy of prediction is shown for groups of bulls whose sires were included (Sire) or whose sires were not included (No Sire) in the training set using trait-specific SNP subsets obtained by PLSR

**Figure 8:**
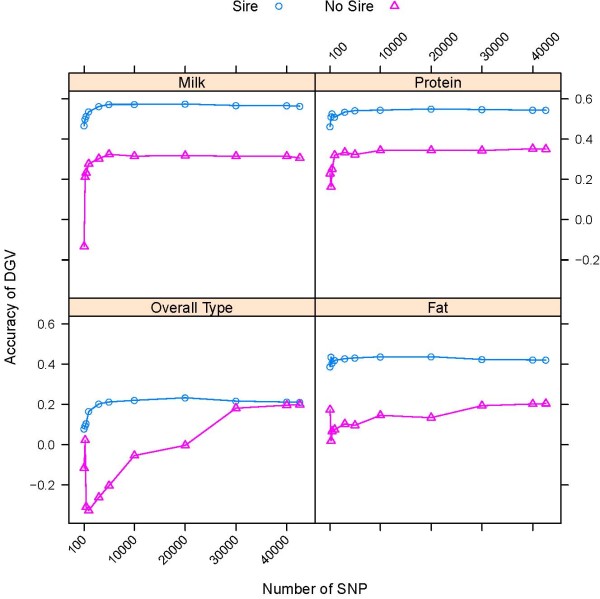
**Accuracy of DGV of cows with or without sire in the training data using trait-specific SNP subsets**. Accuracy of prediction is shown for groups of cows whose sires were included (Sire) or whose sires were not included (No Sire) in the training set using trait-specific SNP subsets obtained by PLSR

Accuracies of DGV predictions of validation animals whose sires were or were not included in the training set were computed from SNP effect estimates obtained by PLSR. As shown in Figure 6, the distribution of additive-genetic relationship differed substantially between validation animals whose sires were or were not represented in the training set. When validation sets were broken up into groups of animals with or without sire in the training data, there was substantial variation in the accuracy of prediction between groups and between bulls and cows (Figure 7 and 8). The number of animals in the group without sire in the training data was small, ranging from 16 to 57 for bulls and from 15 to 37 for cows, depending on the trait. Using the high-density assay, the accuracy of prediction of validation bulls with sire in the training data was not consistently higher than for validation bulls without sire in the training data for all traits (Figure 7). For fat percentage, milk and protein yield, accuracy of prediction when using fewer SNP was consistent between the two groups of bulls, and accuracies varied more for the other traits. However, for cows, the accuracy of DGV for the group whose sire was included in the training data was substantially higher compared to cows without sire in the training data, irrespective of the number of SNP (Figure 8).

## Discussion

The objective of the study was to evaluate the use of low-density SNP assays for genomic selection of dairy cattle. As also shown by Weigel et al. [5] for a single trait, the accuracy of DGV decreased with decreasing number of SNP in the subsets. However, a low-density assay comprising selected SNP can deliver a substantial portion of the gain of a high-density assay, even if a common set of SNP is used across traits. Our results show small differences between RR and PLSR when using high-density assays, but differences between the two methods become more evident for subsets containing fewer SNP.

Recently, a number of studies have reported on the accuracy of DGV for dairy traits [16-18,20,29-32]. These have shown that the accuracy of DGV depends on the size of the training data, SNP density, heritability and the genetic relationships between animals in the training and validation data. Although it is difficult to compare accuracies between studies, accuracies estimated in the current study are within the range of those reported previously.

There was a strong relationship between the accuracy of prediction and the heritability of the trait, with the prediction for production traits, which had with a higher heritability, being more accurate than that for traits with a low heritability. The generally low accuracies of DGV for survival are perhaps in part due to its low heritability (h^2 ^= 0.03, [27]) and the low number of effective records contributing to the DRBV for young bulls (Table 1). For a trait with a low heritability, achieving an accuracy similar to that obtained for production traits requires more records [18,33,34]. Results for the overall type trait were less consistent across the various analyses, with larger differences between bulls and cows and between subset selection strategies compared to other traits. The differences between cows and bulls for overall type can be partly attributed to the fact that the cow's phenotype is derived from a single observation, and the smaller number of animals in the training and validation sets may be responsible for some of the variation between methods of SNP selection. In general, the estimated accuracies reported herein most likely underestimate the correlation between DGV and true breeding value, as the phenotypes (DRBV, DTD and TD) are not perfectly predicting the true breeding value.

Both, RR and PLSR performed very similar in predicting DGV and differences were generally small. However, the highest accuracy of prediction of PLSR was obtained with subsets including considerably fewer SNP than the high-density assay and fewer SNP than the best subset for RR. This might indicate that using PLSR is less appropriate when analysing very large numbers of SNP, although the differences between the maximum accuracy of DGV and the accuracy obtained with 42,576 SNP was small. A similar result has been found by Solberg et al. [9] who have compared PLSR and BayesB for different maker densities in simulated data and found that BayesB gives higher accuracies than PLSR and that the largest difference is obtained with high marker densities. In other simulation studies, Meuwissen et al. [6] and Habier et al. [15] have found higher accuracies for BayesB compared to RR. In all three simulation studies, a limited number of QTL with large effects accounts for most of the genetic variance. This situation is similar to the distribution of QTL effects for fat percentage, where a mutation in the gene DGAT1 [35] is segregating which accounts for 30% of the genetic variance in our population. Of the 300 highest ranked SNP for fat percentage, 11 were located on BTA14 in the region of DGAT1, with the SNP with rank 1 closest to the known mutation. The highest accuracy for fat percentage was obtained with subsets including substantially less SNP than the high-density assay and this suggests that part of the advantage of BayesB over PLSR and RR in the simulations stems from the fact that it simultaneously performs shrinkage of marker coefficients and marker selection [34].

Comparisons of accuracies across traits between validation sets of cows and bulls were constrained by the fact that for cows the accuracy of DGV prediction, computed as the correlation between DGV and DRBV, could not be calculated for five out of the nine traits, as DRBV information was not available for cows. A possible remedy would be to use the correlation between DGV and estimated breeding value, r(DGV, EBV), as a measure of accuracy instead. When we computed r(DGV, EBV) in bulls and cows (results not shown) we found higher values of r(DGV, EBV) in cows than in bulls, and using r(DGV, EBV) would considerably overestimate the accuracy of genomic selection of cows. The higher correlation between DGV and EBV in cows is probably due to a larger contribution of shared information to the EBV through pedigree relationships.

Pedigree relationships between training and validation animals might also have contributed to the accuracy of DGV in cows, with 473 (92.7%) of cows having their sire in the training set compared to 80.8% of the young bulls, and the training set containing more sires of cows (N = 164) than sires of young bulls (N = 30). This could partly explain why for protein, fat and milk yield, the r(DGV, TD) of cows was similar in size as the r(DGV, DTD) for bulls, although the phenotypes of bulls are derived from many more daughter records than the cow phenotypes. Furthermore, the records of cows are included in the sire phenotype. However, the effect of cows contributing information to the sire phenotype is expected to be small, with bulls in the training set which sired a cow in the validation set having on average 5,259 phenotyped daughters. A larger variance of the phenotypes for cows compared to the pre-selected bull teams (Figure 1) has also positively contributed to the correlation between DGV and phenotypes for cows. In addition, the cows were selected from a repository of animals which have been well recorded, so the heritabilities in this subset are most likely higher than in the wider industry.

Habier et al. [16] have demonstrated that the maximum of the additive-genetic relationships between training and validation animals is a good indicator for the accuracy of DGV. The additive-genetic relationship did differ for bulls and cows whose sires were or were not represented in the training set, as shown in Figure 6. As part of the genetic relationship can be captured by SNP [15], one would expect higher accuracies for animals whose sire is included in the training set [5,16]. Our results are less conclusive, as accuracies of DGV for bulls whose sire was included in the training set was not higher than those for bulls whose sire was not included in the training set for all traits. A partial explanation might be the relatively small number of bulls whose sire was not genotyped, resulting in a large sampling error of the correlation between DGV and phenotype. However, an important observation is that the differences in accuracies between the two groups appear to be independent of the number of SNP and the method of SNP selection [see Additional files 2 and 3], with the exception of survival and overall type and possible reasons for this are discussed above.

In practise, whilst an attractive application of genotyping using low-density assays is the selection of replacement heifers, for reasons given above, the accuracy is expected to be smaller than reported here for cows. However, the accuracy of genomic selection will be increased if DGV predictions are combined with information from pedigree [17,18,20].

Few SNP were in common between the trait-specific subsets (Figure 5) and, given that at least 1,000 SNP are required to obtain accurate DGV predictions for most traits, combining the highest ranked SNP for each trait onto a single chip or developing multiple low-density assays might not provide adequate reductions in genotyping costs. Irrespective of the method of SNP selection, subsets of 3,000 evenly spaced SNP provided more than 90% of the accuracy that can be achieved with a high-density assay in genomic selection of cows and 80% of the high-density assay in young bulls. Furthermore, the rate of increase in accuracy with increasing size of the subset was more rapid for evenly spaced SNP, so that the additional gain from using trait-specific assays or SNP related to a single index such as ASI or APR was small for subsets with a larger number of SNP.

Predictions using subsets including 3,000 of the highest ranked SNP were only 1.06 times more accurate in bulls and 1.03 times more accurate in cows than a common subset of evenly spaced SNP of the same size selected based on MAF. This suggests that the distribution of true effects is more or less spread among many loci across the genome and that 3,000 evenly spaced SNP largely capture the level of LD present in the population. While accuracies based on at least ~1,000 or 3,000 SNP appear to be very robust to the method used to select those SNP, accuracies are very sensitive when fewer than 1,000 SNP are used (Figure 3 and 4). However, without re-estimation of effects, accuracy when using evenly spaced low-density assays is expected to decrease steadily and faster over generations compared to accuracy from a high-density assay or from subsets including SNP with large effects, but this loss could be limited by genotyping the parents used for breeding with the high-density assay and retraining [36]. Dense genotyping of some parents might not be necessary if the genotype information of the high-density assay can be imputed from a low-density SNP panel [36,37].

Weigel et al. [5] have also assessed the ability to predict DGV using subsets of SNP with largest effects and subsets of evenly spaced SNP for the trait lifetime net merit in dairy cattle. Although it is difficult to compare accuracies between studies due to differences in the methods used to calculate DGV, the size of the training data and the number of SNP of the high-density assay, both studies agree well in that a trait-specific subset including 2,000 of the highest ranked SNP captured most of the gain achieved with a high-density assay. However, for subsets of evenly spaced SNP, the rate in loss of accuracy with decreasing SNP numbers was lower in our study compared to [5]. Here, selection of SNP was performed by choosing the highest ranked SNP within segments of approximately equal size, whereas in [5] spacing of SNP was only informed by the position of the SNP. Using the latter approach, one would expect a larger number of low ranked SNP to be selected in the assay. Indeed, when compared to subsets including evenly spaced SNP selected for APR, we found that selecting SNP only on their position reduced the relative accuracy between 5% and 17% depending on the size of the subset and the trait analysed (results not shown).

Although ranking of SNP was based on the magnitude of the estimated SNP effects, SNP selected on their rank had a higher average minor allele frequency (*p*_MAF_) than all SNP on the high-density assay. For example, subsets containing 300 evenly spaced SNP selected for ASI by PLSR had a mean *p*_MAF _= 0.33 compared to *p*_MAF _= 0.30 for SNP selected by RR and *p*_MAF _= 0.27 for SNP selected on only their location. This suggests that selection of SNP should be based on their expected contributions to the genetic variance, which is a function of the allele frequency in the training set. This also implies that accuracy of prediction will be lower for a validation set where the distribution of allele frequencies does not resemble that of the training set and why prediction equations derived in one breed do not predict accurate DGV when applied to other breeds, as shown by Hayes et al. [31].

Arguably, the major advantage of a low cost SNP assay will be that training sets will become much larger, as relatively more animals are genotyped and hence accuracy of DGV will increase (e.g.[38]). The current reference populations predominantly consist of elite progeny tested sires and to significantly increase the size of the training data will require the genotyping of cows.

## Conclusions

Genomic selection has become a routine in dairy cattle breeding programs worldwide. The current cost of whole-genome selection based on dense SNP genotypes has limited the application to the selection of elite males and females that are likely to become parents of the next generation. Results of our study indicate that accurate genomic evaluation of the broader bull and cow population can be achieved with genotyping assays containing ~ 3,000 to 5,000 SNP. A chip containing 3,000 evenly spaced markers can provide approximately 90% of the accuracy achieved with a high-density SNP assay for genomic selection of bulls and cows combined across traits. Possible applications include the selection of replacement heifers and the pre-screening of young bulls and potential bull dams. Assays with evenly spaced markers are preferable as they can be used across traits and possibly across populations. It also allows for a high volume generic chip to be produced, which will lower assay cost per individual and will limit heterogeneity of genomic information compared to using multiple assays for different traits. Evenly spaced low-density assays might also permit the reconstruction of the genotype information of high-density assays through imputation, which is important in situations where, for example, high-density genotyping is limited to nucleus breeding herds. Increasing the proportion of animals genotyped will further increase the accuracy of genomic selection as the training data grows over time, particularly through genotyping of cows.

## Competing interests

The authors declare that they have no competing interests.

## Authors' contributions

GM was the principal investigator in the design of the study and methods, carried out the statistical analysis and drafted the manuscript. MSK and BJH participated in the analysis, had a role in data acquisition, assembly and data QC and contributed to the manuscript preparation. HWR contributed to project design, data acquisition, and contributed to the manuscript preparation. All authors read and approved the final manuscript.

## Supplementary Material

Additional file 1 **Accuracy of DGV of bulls and cows using subsets of 5,000 or less of the highest ranked SNP obtained by RR and PLSR**. Enlarged representation of Figure 2 for subsets of up to 5,000 SNP to make differences between RR and PLSR more visible Click here for file

Additional file 2 **Accuracy of DGV of bulls whose sires were included (Sire) or were not included (No Sire) in the training set depending on the method of SNP selection**. Accuracy of prediction is shown for subsets including the highest ranked SNP (Trait), subsets of evenly spaced SNP including the highest ranked SNP for ASI (ASI), APR (APR) or SNP with highest minor allele frequency (MAF) obtained by PLSR Click here for file

Additional file 3 **Accuracy of DGV of cows whose sires were included (Sire) or were not included (No Sire) in the training set depending on the method of SNP selection**. Accuracy of prediction is shown for subsets including the highest ranked SNP (Trait), subsets of evenly spaced SNP including the highest ranked SNP for ASI (ASI), APR (APR) or SNP with highest minor allele frequency (MAF) obtained by PLSR Click here for file
