# Supplementary figures and images for: Accuracy of direct genomic values in Holstein bulls and cows using subsets of SNP markers
Source: Genet Sel Evol. 2010 Oct 16;42(1):37. doi: 10.1186/1297-9686-42-37 (PMC2964565; doi:10.1186/1297-9686-42-37)

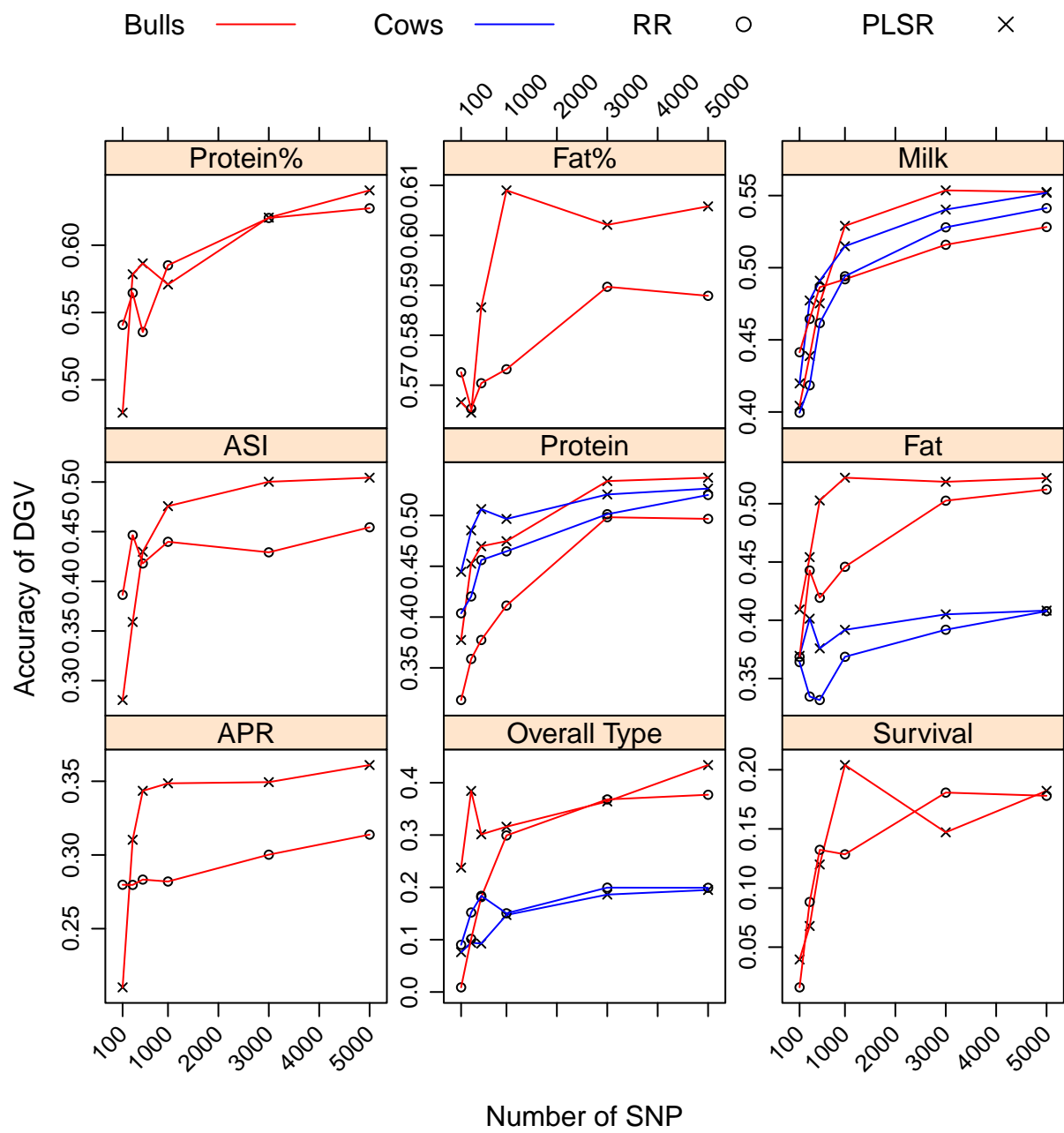

Supplement: Additional file 1 — Accuracy of DGV of bulls and cows using subsets of 5,000 or less of the highest ranked SNP obtained by RR and PLSR. Enlarged representation of Figure 2 for subsets of up to 5,000 SNP to make differences between RR and PLSR more visible [file 1297-9686-42-37-S1.PDF]

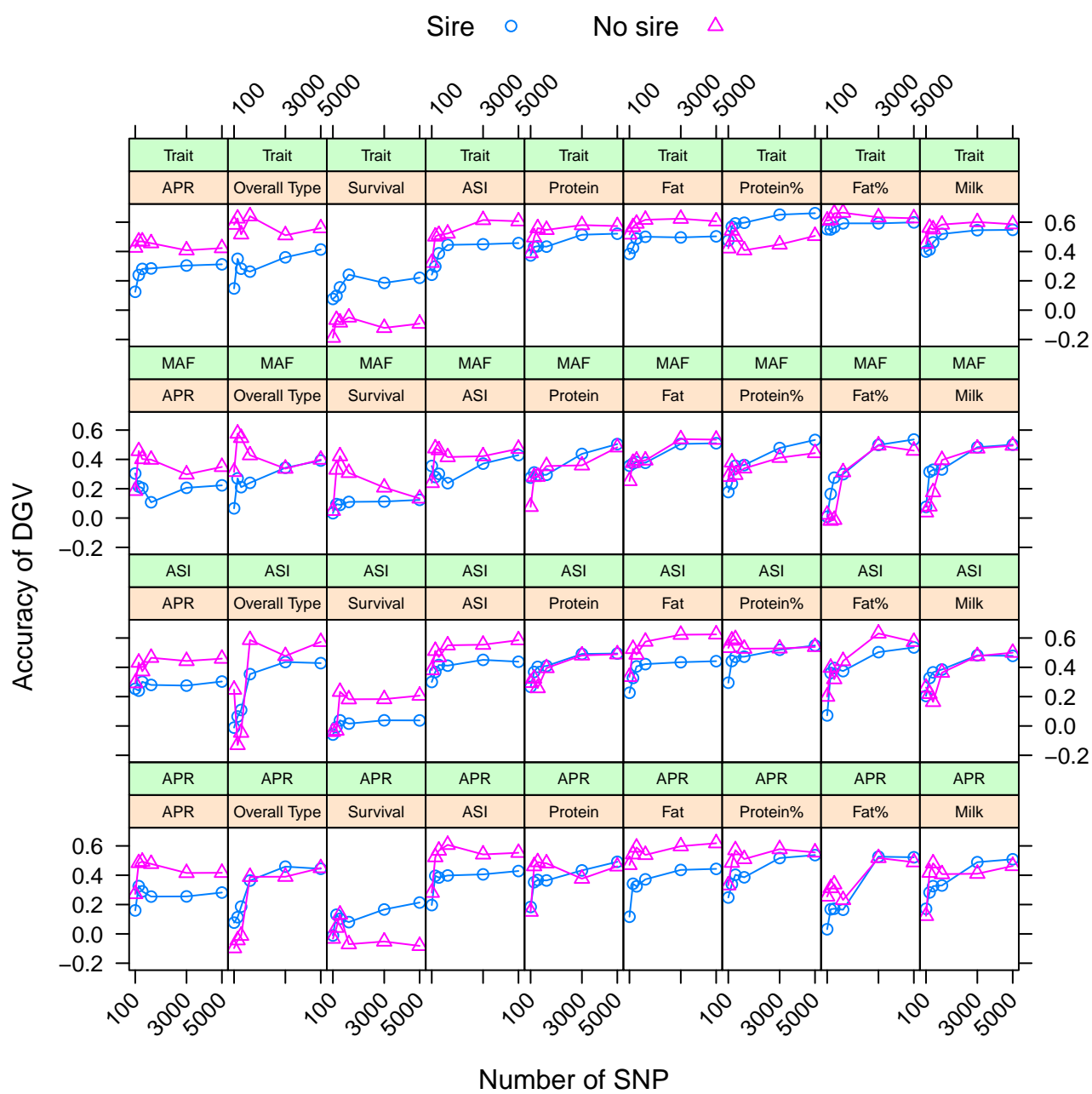

Supplement: Additional file 2 — Accuracy of DGV of bulls whose sires were included (Sire) or were not included (No Sire) in the training set depending on the method of SNP selection. Accuracy of prediction is shown for subsets including the highest ranked SNP (Trait), subsets of evenly spaced SNP including the highest ranked SNP for ASI (ASI), APR (APR) or SNP with highest minor allele frequency (MAF) obtained by PLSR [file 1297-9686-42-37-S2.PDF]

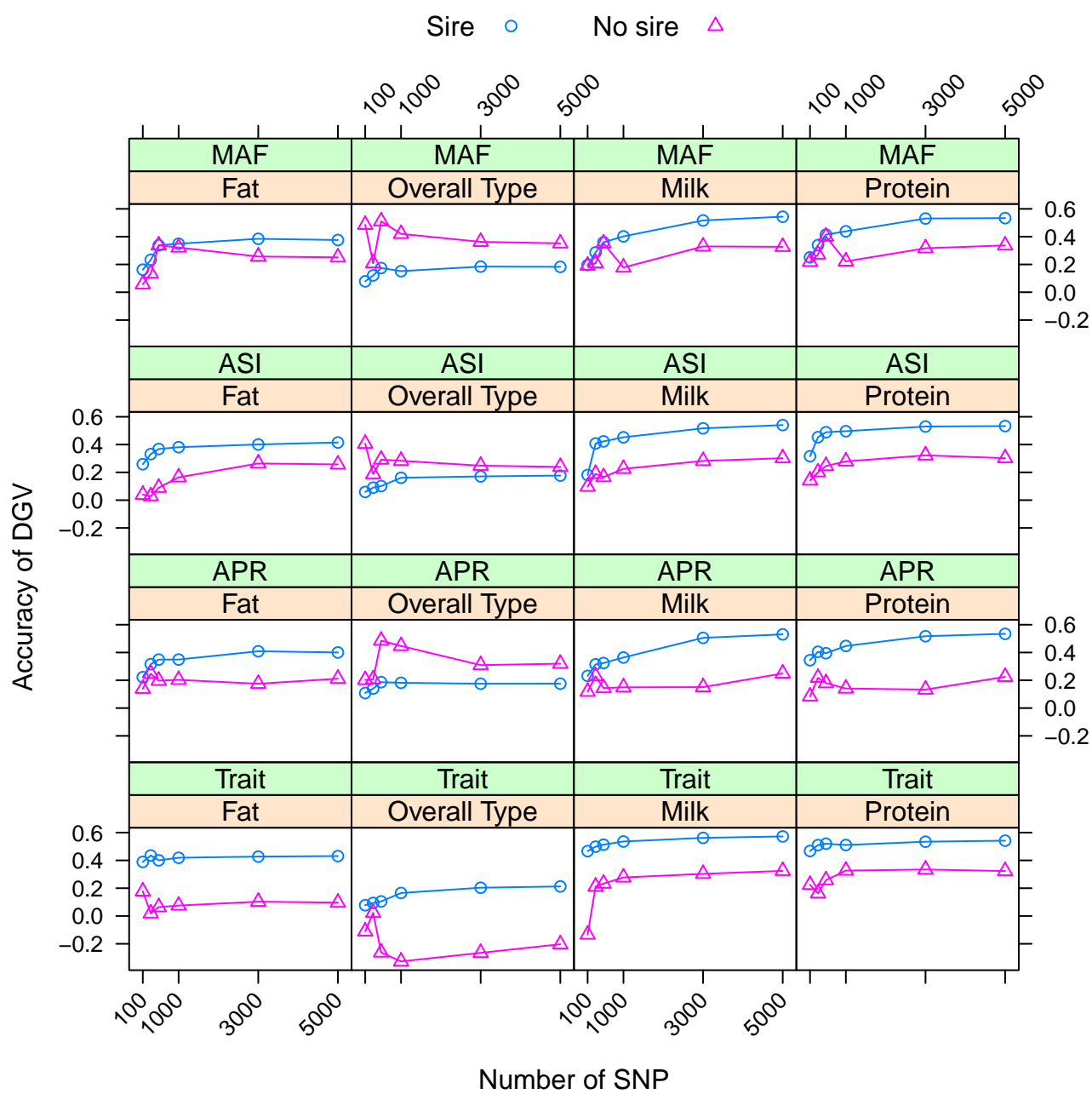

Supplement: Additional file 3 — Accuracy of DGV of cows whose sires were included (Sire) or were not included (No Sire) in the training set depending on the method of SNP selection. Accuracy of prediction is shown for subsets including the highest ranked SNP (Trait), subsets of evenly spaced SNP including the highest ranked SNP for ASI (ASI), APR (APR) or SNP with highest minor allele frequency (MAF) obtained by PLSR [file 1297-9686-42-37-S3.PDF]
